# Immediate CT after hospital arrival and decreased in-hospital mortality in severely injured trauma patients

**DOI:** 10.1093/bjsopen/zrac133

**Published:** 2023-01-21

**Authors:** Ryo Yamamoto, Masaru Suzuki, Tomohiro Funabiki, Junichi Sasaki

**Affiliations:** Department of Emergency and Critical Care Medicine, Keio University School of Medicine, Tokyo, Japan; Department of Emergency Medicine, Tokyo Dental College, Ichikawa General Hospital, Chiba, Japan; Department of Emergency Medicine, Fujita Health University Hospital, Aichi, Japan; Department of Emergency and Critical Care Medicine, Keio University School of Medicine, Tokyo, Japan

## Abstract

**Background:**

Immediate whole-body CT (about 10 min after arrival) in an all-in-one resuscitation room equipped with CT has been found to be associated with shorter time to haemostasis and lower in-hospital mortality. The aim of this study was to elucidate the benefits of immediate whole-body CT after hospital arrival in patients with severe trauma with the hypothesis that immediate CT within 10 min is associated with lower in-hospital mortality.

**Method:**

This retrospective cohort study of patients with an injury severity score of more than 15 who underwent whole-body CT was conducted using the Japanese Trauma Databank (2019–2020). An immediate CT was conducted within 10 min after arrival. In-hospital mortality, frequency of subsequent surgery, and time to surgery were compared with immediate and non-immediate CT. Inverse probability weighting was conducted to adjust for patient backgrounds, including mechanism and severity of injury, prehospital treatment, vital signs, and institutional characteristics.

**Results:**

Among the 7832 patients included, 646 underwent immediate CT. Immediate CT was associated with lower in-hospital mortality (12.5 *versus* 15.7 per cent; adjusted OR 0.77 (95 per cent c.i. 0.69 to 0.84); *P* < 0.001) and fewer damage-control surgeries (OR 0.75 (95 per cent c.i. 0.65 to 0.87)). There was a 10 to 20 min difference in median time to craniotomy, laparotomy, and angiography. These benefits were observed regardless of haemodynamic instability on hospital arrival, while they were identified only in elderly patients with severe injury and altered consciousness.

**Conclusion:**

Immediate CT within 10 min after arrival was associated with decreased in-hospital mortality in severely injured trauma patients.

## Teaser

Immediate whole-body CT (about 10 min after arrival) in an all-in-one resuscitation room equipped with CT was associated with shorter time to haemostasis and lower in-hospital mortality; however, the generalizability of benefits from rapid whole-body CT is inconclusive. The present study analysed a nationwide trauma registry and identified that immediate CT within 10 min after arrival was associated with decreased in-hospital mortality in severely injured trauma patients.

## Introduction

CT for trauma patients can detect diverse injuries that require early intervention^1^. While physical assessment typically determines the need for CT and the regions to scan, whole-body head-to-pelvis CT, rather than selected-region CT, is often performed in patients with a high-risk injury mechanism, altered consciousness, or severe pain^2,3^. Although discussion is ongoing regarding indications for whole-body CT, obtaining CT images of multiple regions immediately after a quick physical evaluation has become more frequent for trauma patients wordwide^4,5^.

As conducting CT without appropriate life-saving procedures introduces unfavourable clinical outcomes^6,7^, all-in-one resuscitation rooms equipped with CT have been developed to perform imaging tests safely and shorten the door-to-CT time^8^. A study of trauma resuscitation in these rooms reported that immediate whole-body CT after arrival, even before physical assessment for haemorrhage, was associated with shorter time to haemostatic procedures and lower in-hospital mortality in severe trauma^9,10^; however, as harm from haemodynamic instability during the scan outside the resuscitation rooms may negate the beneficial effects of early haemostasis^11,12^, the generalizability of benefits from rapid whole-body CT is inconclusive.

Accordingly, using a nationwide trauma database, the aim of this study was to examine the outcomes of whole-body CT in severely injured trauma patients to elucidate the benefits and identify optimal candidates for immediate CT after hospital arrival. Whole-body CT performed within 10 min after arrival was considered as an immediate CT based on a previous study^9^ and the hypothesis was that immediate CT would be associated with lower in-hospital mortality in patients with severe trauma.

## Methods

### Study design and setting

This retrospective cohort study used the Japan Trauma Data Bank (JTDB), a nationwide trauma registry established in 2003 (*Appendix S1*). The JTDB has been maintained by the Japanese Association for the Surgery of Trauma (JAST) and the Japanese Association for Acute Medicine, representing more than 250 participating hospitals. There are approximately 300 high-level tertiary care centres in Japan and most participate in the JTDB^13^. All collaborating hospitals obtained individual local institutional review board approval for human research before initiating this study. This study was approved by the Keio University School of Medicine Institutional Review Board (application number: 20090087). Informed consent was waived because of the anonymous nature of the data.

In Japan, the resuscitation of trauma patients usually follows a management protocol developed by the JAST based on the Advanced Trauma Life Support (ATLS) training programme of the American College of Surgeons. CT is generally recommended as an adjunct examination after physiological assessment for life-threatening injuries and resuscitative procedures such as airway management, transfusion, and emergency haemostatic surgery^14^. While there is no clear indication for head-to-pelvis whole-body CT, patients with a high-energy injury, injuries in multiple regions, and altered consciousness likely undergo whole-body CT in most institutions.

Although CT for haemodynamically unstable patients is not recommended in the JAST protocol, CT with simultaneous airway and respiratory management immediately after hospital arrival is often conducted in Japan, particularly when obtaining CT images is not expected to delay haemostatic surgery. The attending physician decides to transfer hypotensive patients to a CT room, usually based on human resources for simultaneous resuscitation in a CT room and the time to prepare the operating room.

### Study population

Data from the JTDB between 2019 and 2020 were retrospectively reviewed. Adult trauma patients (18 years and older) who underwent whole-body CT, and were directly transported from the scene were included. Patients with an injury severity score (ISS) less than 15 and those who underwent CT later than 1 h after hospital arrival were excluded because they were not considered as severely injured trauma patients.

### Data collection and definitions

Prehospital data were collected by emergency medical service personnel and in-hospital by each institution’s treating physician. The information from the database included age, sex, Charlson co-morbidity index, mechanism of injury, vital signs on arrival, lactate value on arrival, abbreviated injury scale (AIS) score, ISS, revised trauma score, prehospital procedures, including manual ventilation, intubation, and fluid administration, the presence of a physician prehospital, transportation time, haemostatic procedures, including angiography and surgery, time to the haemostatic procedure from hospital arrival, length of intensive care unit (ICU) and duration of hospital stay, Glasgow outcome scale (GOS) at hospital discharge, and survival status at discharge. The whole-body CT indication, haemodynamic status before, during, and after CT, and details of physiological findings during the initial assessment were not available in the database.

Immediate CT was defined as CT conducted within 10 min after hospital arrival (door-to-CT time of 10 min or less); a non-immediate CT was defined as a CT performed 10–60 min after arrival. The frequency of immediate CT at each institution was calculated. Participating hospitals were categorized into three different immediate CT rates using cut-off values that trisect the number of patients as equally as possible: low, moderate, and high. Delayed hospital arrival was a transportation time greater than 30 min.

### Outcome measures

The primary outcome was in-hospital mortality. Secondary outcomes included neurological status (measured by GOS) at discharge, length of ICU and duration of hospital stay, and the need for damage-control surgery, haemostatic surgery, angiography, and time to the haemostatic procedure from hospital arrival.

### Statistical analysis

The primary outcome was compared between patients with immediate and non-immediate CT. Inverse probability weighting (IPW) with propensity scores was performed to adjust covariates by weighting between patients with and without an immediate CT^15^, by which the primary and secondary outcomes were compared. The propensity score was developed using a logistic regression model to estimate the probability of conducting immediate CT^16^. Relevant covariates were carefully selected from known or possible predictors for performing immediate CT and predicting clinical outcomes in severe trauma patients based on previous studies^17^, including age, sex, Charlson co-morbidity index, mechanism of injury, the severity of injuries, and vital signs on hospital arrival (systolic blood pressure (SBP), heart rate, respiratory rate, and Glasgow coma scale (GCS)), delayed hospital arrival, and the frequency of immediate CT at each institution^13,18,19^. Prehospital procedures, including oxygen and fluid administration, and the presence of a physician prehospital^20^, were also included as covariates as they are survival predictors in trauma patients that are not affected by door-to-CT time. Patients with missing covariates were excluded from the propensity score calculation. The discrimination and calibration precision of the propensity score were analysed using the c-statistic and the Hosmer–Lemeshow goodness-of-fit test^16^. The IPW analyses were performed as adjusted analyses in which the primary outcome was compared using the chi-squared test^15^. Secondary outcomes were evaluated with ORs or median differences using the Hodges–Lehmann estimator.

Sensitivity analyses were performed to validate the primary results. Multivariate logistic regression was conducted with the forward stepwise method based on the probability of the Wald statistic using covariates for the propensity score calculation to confirm that results were not dependent on the propensity score. In addition, considering differences in quality of care between participating institutions, a generalized estimating equation analysis with logit link function using the same covariates for propensity score development was performed^21^. Moreover, to avoid extreme weight by propensity scores, IPW with restriction was performed after excluding patients with a propensity score of 0.05 or less or 0.95 or greater^15^.

Restricted cubic spline curves for estimating in-hospital mortality by door-to-CT time were created to examine whether the cut-off for defining immediate CT was appropriate. For this, a multivariate logistic regression model with the stepwise method was adopted.

Subgroup analyses examined the relationships between immediate CT, clinical characteristics, and in-hospital mortality. Targeted subgroups were selected based on previous studies that investigated clinical outcomes of trauma patients^19,22,23^. The IPW analyses on the primary outcome were repeated in the patient subgroups divided by age (less than 65 *versus* 65 years or older), the severity of injury (ISS less than 25 *versus* 25 or higher), haemodynamic instability on hospital arrival (SBP 90 mmHg or higher *versus* less than 90 mmHg), and level of consciousness on hospital arrival (GCS 13 or higher *versus* 12 or less). Subgroup analyses were also performed in divided populations based on intubation during resuscitation and the frequency of immediate CT (rate of immediate CT 5 per cent or higher *versus* less than 5 per cent).

Descriptive statistics are presented as a median (interquartile range (i.q.r.)) or a number (percentage). Results are shown using the standardized difference and the 95 per cent confidence interval (c.i.); a standardized difference less than 0.1 was considered insignificant^16^. The hypothesis was only tested on the primary outcome in which a two-sided α threshold of 0.05 was considered significant. Based on the previous study, 9 *versus* 14 per cent mortality was expected in patients with immediate and non-immediate CT respectively, and sample size calculation with 0.05 of α error and 0.8 of power suggested at least 638 cases in each group would be needed for the main analysis. All statistical analyses were conducted using SPSS^®^ Statistics for Windows version 27.0 (IBM, Armonk, NY, USA) and Microsoft^®^ Excel (Microsoft, Redmond, WA, USA).

## Results

### Patient characteristics

**Fig. 1:**
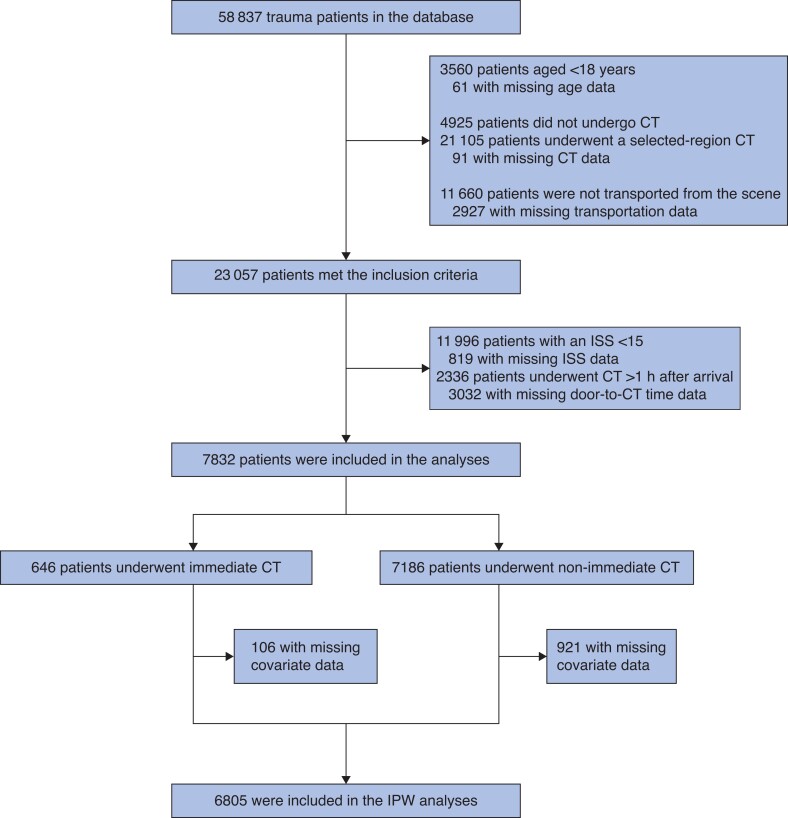
Patient flow diagram Among 58 837 trauma patients in the database, 7832 severely injured adults were transported directly from the scene and underwent whole-body CT within 1 h after arrival; therefore, they were eligible for this study. A total of 106 patients with immediate CT and 921 patients with non-immediate CT were excluded from IPW analyses because of missing covariates for the propensity score; hence, IPW analyses were performed for 6805 patients. IPW, inverse probability weighting; ISS, injury severity score.

Of the 58 837 trauma patients in the database, 7832 severely injured adults were transported from the scene by ambulance and underwent whole-body CT within 1 h after hospital arrival and were therefore eligible for this study (*Fig. 1*).

In total, 646 (8.2 per cent) patients underwent CT within 10 min after arrival. Patient characteristics are shown in *Table 1*. The median door-to-CT time was 7 min in immediate CT and 26 min in non-immediate CT. Patients with an immediate CT (door-to-CT of 10 min or less) had a higher SBP on arrival, higher ISS, and higher AIS in the head compared with those with non-immediate CT. Additionally, a greater number of patients with immediate CT were seen by a physician before hospital and received fluids before hospital arrival.

**Table 1:** Characteristics of trauma patients with whole-body CT

	Before IPW			After IPW		
Characteristic	Immediate CT	Non-immediate CT	Standardized difference	Immediate CT	Non-immediate CT	Standardized difference
n	646	7186	–	–	–	–
Door-to-CT time (min), median (i.q.r.)	7 (5–9)	26 (19–36)	2.498	7 (6–9)	26 (19–36)	2.444
Age (years), median (i.q.r.)	68 (50–77)	66 (47–77)	0.070	65 (50–77)	66 (47–77)	0.001
Sex (male)	457 (71.3)	5082 (71.1)	0.005	4722 (70.5)	4883 (71.8)	0.028
Mechanism of injury (blunt)	626 (98.7)	6934 (98.8)	0.006	6656 (99.4)	6718 (98.7)	0.063
Charlson co-morbidity, index, median (i.q.r.)	0 (0–1)	0 (0–1)	0.000	0 (0–0)	0 (0–1)	0.085
**Vital signs on arrival, median (i.q.r.)**						
GCS	14 (7–15)	14 (7–15)	0.051	14 (10–15)	14 (8–15)	0.069
SBP (mmHg)	135 (109–159)	130 (104–156)	0.103	123 (100–152)	131 (105–157)	0.090
HR (per min)	84 (70–99)	83 (69–100)	0.067	82 (68–99)	82 (69–99)	0.041
RR (per min)	20 (17–24)	20 (17–24)	0.024	20 (18–24)	20 (17–25)	0.017
SpO_2_ (%)	99 (97–100)	99 (96–100)	0.018	99 (96–100)	99 (96–100)	0.060
BT (°C)	36.3 (35.8–36.7)	36.3 (35.8–36.7)	0.039	36.3 (35.8–36.8)	36.3 (35.8–36.7)	0.014
Lactate, ≥ 2 (mmol/l)	217 (52)	3316 (60.8)	0.177	3111 (66)	3083 (59.6)	0.023
**ISS**						
17–19	99 (15.3)	1133 (15.8)	0.012	891 (13.3)	1102 (16.2)	0.082
20–23	67 (10.3)	939 (13.1)	0.084	706 (10.5)	915 (13.4)	0.090
24–27	160 (24.8)	1417 (19.7)	0.122	1161 (17.3)	1352 (19.9)	0.065
28–43	159 (24.6)	1622 (22.6)	0.048	1686 (25.2)	1577 (23.2)	0.046
**AIS, median (i.q.r.)**						
Head	3 (0–4)	2 (0–4)	0.174	2 (1–4)	2 (0–4)	0.000
Face	0 (0–0)	0 (0–0)	0.014	0 (0–1)	0 (0–0)	0.068
Neck	0 (0–0)	0 (0–0)	0.056	0 (0–0)	0 (0–0)	0.031
Chest	0 (0–3)	1 (0–3)	0.036	2 (0–3)	1 (0–3)	0.012
Abdomen/pelvis	0 (0–0)	0 (0–0)	0.037	0 (0–0)	0 (0–0)	0.000
RTS	7.84 (5.97–7.84)	7.84 (5.97–7.84)	0.039	7.55 (6.17–7.84)	7.84 (5.97–7.84)	0.006
**Prehospital procedure**						
Manual ventilation	25 (3.9)	377 (5.2)	0.066	243 (3.6)	293 (4.3)	0.035
Intubation	25 (3.9)	194 (2.7)	0.066	174 (2.6)	156 (2.3)	0.020
Fluid administration	116 (18.0)	979 (13.6)	0.119	925 (13.8)	921 (13.5)	0.008
Physician presence before hospital	326 (50.5)	1387 (19.3)	0.692	1488 (22.2)	1470 (21.6)	0.015
Transportation time (min), median (i.q.r.)	14 (9–21)	12 (8–20)	0.021	13 (8–20)	13 (8–20)	0.022
Delayed arrival	76 (12.5)	726 (10.5)	0.063	725 (10.8)	742 (10.9)	0.003
**Frequency of immediate CT at institution**						
Low (<1.85%)	12 (1.9)	2245 (31.2)	0.861	1650 (24.6)	1950 (28.7)	0.091
Moderate (1.85–4.85%)	85 (13.2)	2569 (35.8)	0.545	2547 (38.0)	2329 (34.2)	0.079
High (>4.85%)	549 (85)	2372 (33.0)	1.245	2501 (37.3)	2525 (37.1)	0.005

Values are *n* (%) unless otherwise indicated. IPW, inverse probability weighting; i.q.r., interquartile range; GCS, Glasgow coma scale; SBP, systolic blood pressure; HR, heart rate; RR, respiratory rate; BT, body temperature; ISS, injury severity score; AIS, abbreviated injury scale; RTS, revised trauma score.

A propensity model predicting whether to undergo immediate CT was developed, and discrimination and calibration were calculated: c-statistic = 0.844 (0.829–0.859) and Hosmer–Lemeshow goodness of fit, *P* = 0.450. In total, 106 patients with immediate CT and 921 patients with non-immediate CT were excluded from IPW analyses because of missing covariates for the propensity score; therefore, IPW analyses were performed for 6805 patients (*Fig. 1*). *Table 1* shows the patient characteristics after IPW with standardized differences, wherein differences in covariates, including prehospital resuscitation, vital signs on arrival, AIS, and ISS, were successfully attenuated using the propensity score (standardized difference < 0.1). Variance ratios of continuous variables after IPW are shown in *Table S1* and box-plots of the adjusted and non-adjusted propensity scores are shown in *Fig. S1*.

### In-hospital mortality and secondary outcomes

**Fig. 2:**
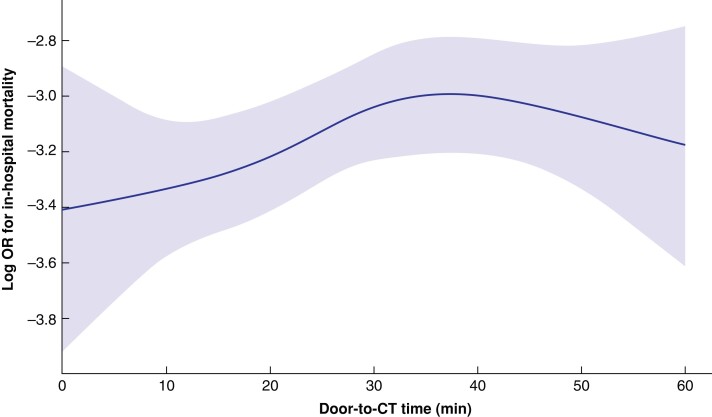
Restricted cubic spline curve of mortality prediction by door-to-CT time Restricted cubic spline curves for estimating in-hospital mortality by door-to-CT time were drawn, in which a multivariate logistic regression model with the stepwise method was adopted. A linear decrease of hazard of death by shortening the door-to-CT time was observed under the condition that CT was conducted within 30–40 min after hospital arrival.

In-hospital mortality among patients with an immediate CT was significantly lower than among those with a non-immediate CT (12.5 *versus* 15.7 per cent; OR 0.77 (95 per cent c.i. 0.69 to 0.84); *P* < 0.001) (*Table 2*). Sensitivity analyses with multivariate logistic regression and generalized estimating equations showed similar findings; immediate CT was associated with reduced in-hospital mortality in severe trauma patients (OR 0.67 (95 per cent c.i. 0.46 to 0.97) and 0.69 (95 per cent c.i. 0.49 to 0.99) respectively) (*Table S2*). In addition, IPW with restriction revealed a relationship between immediate CT and decreased in-hospital mortality (OR 0.81 (95 per cent c.i. 0.69 to 0.93); *Table S2*). Furthermore, the restricted cubic spline curve of mortality prediction by door-to-CT time showed a linear decrease of hazard of death by shortening the door-to-CT time, under the condition that CT was conducted within 30–40 min after hospital arrival (*Fig. 2*).

**Table 2:** Immediate CT and clinical outcomes

Clinical outcome	Immediate CT	Non-immediate CT	*P*	OR (95% c.i.)	Difference in median (95% c.i.)	
In-hospital mortality, % (95% c.i.)	12.5 (11.7–13.3)	15.7 (14.8–16.6)	<0.001	0.77 (0.69–0.84)	–	–
Glasgow outcome scale at discharge, *≥ 4,* % (95% c.i.)	64.0 (62.5–65.5)	55.6 (54.1–57.1)	–	1.42 (1.30–1.55)	–	–
Length of ICU stay (days), median (i.q.r.)	3 (0–8)	3 (0–7)	–	–	0	(0 to 0)
Duration of hospital stay (days), median (i.q.r.)	26 (9–38)	23 (10–41)	–	–	0	(0 to 1)
Damage-control surgery, % (95% c.i.)	8.4 (7.5–9.3)	10.9 (9.9–11.8)	–	0.75 (0.65–0.87)	–	–
Haemostatic surgery, % (95% c.i.)	17.9 (17.0–18.9)	17.1 (16.2–18.0)	–	1.06 (0.97–1.16)	–	–
Angiography, % (95% c.i.)	6.2 (5.6–6.8)	9.5 (8.8–10.1)	–	0.64 (0.56–0.72)	–	–
Time to craniotomy (min), median (i.q.r.)	106 (99–351)	142 (97–237)	–	–	−13	(−26 to −2)
Time to laparotomy (min), median (i.q.r.)	108 (68–232)	134 (83–195)	–	–	−23	(−45 to 4)
Time to angiography (min), median (i.q.r.)	74 (41–148)	95 (64–147)	–	–	−24	(−47 to 0)

i.q.r., interquartile range; ICU, intensive care unit.

Immediate CT was associated with a better neurological outcome (GOS 4 or higher) at hospital discharge (OR 1.42 (95 per cent c.i. 1.30 to 1.55); *Table 2*), but not with the length of hospital or ICU stay. Moreover, the requirement for damage-control surgery and angiography was less frequent in patients with immediate CT compared with non-immediate CT (OR 0.75 (95 per cent c.i. 0.65 to 0.87) and 0.64 (95 per cent c.i. 0.56 to 0.72) respectively); *Table 2*), but the frequency of haemostatic surgery was comparable. Differences of 10–20 min were observed in median time to craniotomy, laparotomy, and angiography between patients with immediate and non-immediate CT, although these differences were not statistically significant.

### Subgroup analysis

In subgroup analyses (*Table 3*), a relationship between decreased in-hospital mortality and immediate CT was observed in several subgroups, patients with and without haemodynamic instability on hospital arrival and those treated at institutions with a lower and higher frequency of immediate CT.

**Table 3:** In-hospital mortality in subgroup analyses

Variable	Immediate CT	Non-immediate CT	OR	95% c.i.
**Age**				
<65 (years)	10.8 (9.6–11.9)	12.1 (11.0–13.2)	0.87	0.75–1.02
≥65 (years)	13.8 (12.7–14.9)	19.0 (17.7–20.3)	0.68	0.60–0.77
**Severity of injury**				
ISS <25	6.1 (5.2–6.9)	5.6 (4.9–6.3)	1.09	0.89–1.34
ISS ≥25	17.3 (16.1–18.5)	29.2 (27.5–30.8)	0.51	0.45–0.57
**Haemodynamic instability on arrival**				
SBP ≥90 (mmHg)	9.3 (8.5–10)	10.5 (9.7–11.3)	0.87	0.77–0.99
SBP <90 (mmHg)	26 (23.6–28.4)	44.8 (41.8–47.9)	0.43	0.36–0.52
**Consciousness on hospital arrival**				
GCS ≥13	4 (3.4–4.7)	3.5 (4.0–4.1)	1.15	0.92–1.45
GCS ≤12	23.9 (22.3–25.5)	39.1 (37.1–41.1)	0.49	0.43–0.55
**Intubation during resuscitation**				
No intubation	7.8 (7.1–8.6)	6.9 (6.2–7.6)	1.15	0.99–1.34
Intubation	26.8 (24.6–28.9)	38.8 (36.6–41.0)	0.58	0.50–0.67
**Frequency of immediate CT**				
<5%	11.6 (10.7–12.6)	15.1 (14.1–16.2)	0.74	0.65–0.84
≥5%	13.9 (12.5–15.3)	16.7 (15.2–18.1)	0.81	0.69–0.94

Values are % (95% CI). IPW analyses were performed in each subgroup. ISS, injury severity score; SBP, systolic blood pressure; GCS, Glasgow coma scale; IPW, inverse probability weighting.

In contrast, younger patients (less than 65 years) and those with ISS less than 25 and GCS of 13 or higher on arrival had comparable mortality regardless of the whole-body CT timing, whereas older patients (65 years or older) and those with severe injury (ISS of 25 or higher) and decreased consciousness on arrival (GCS of 12 or less) had significantly reduced in-hospital mortality with immediate CT (OR 0.68 (95 per cent c.i. 0.60 to 0.77), 0.51 (95 per cent c.i. 0.45 to 0.57), and 0.49 (95 per cent c.i. 0.43 to 0.55) respectively).

In addition, an association between immediate CT and lower mortality was observed in patients who were intubated during the initial resuscitation, but not in those who were not intubated (OR 0.58 (95 per cent c.i. 0.50 to 0.67) and 1.15 (95 per cent c.i. 0.99 to 1.34) respectively).

## Discussion

This study revealed that immediate CT with a door-to-CT time of 10 min or less was associated with lower in-hospital mortality and better neurological outcomes than non-immediate CT after adjustment for patient characteristics. This result was also consistent across several sensitivity analyses, although causality between immediate CT and favourable outcomes cannot be demonstrated in this study and should be validated in future studies.

One advantage of CT immediately after hospital arrival in severe trauma patients would be an early diagnosis of injuries that require therapeutic intervention^24^. Retrospective studies of trauma patients resuscitated at an all-in-one resuscitation room equipped with CT identified a significantly shorter door-to-CT time, time to surgery, and increased survival, compared with historical controls^9,10^. Similar results were identified in this study; about a 20-min shortening of door-to-CT time and a possible 10–20-min reduction in time to craniotomy, laparotomy, and angiography were observed in patients who underwent immediate CT after hospital arrival. Moreover, early therapeutic intervention after immediate CT may increase the chances of achieving definitive haemostasis before coagulopathy emerges. The duration until haemostasis and amount of bleeding were associated with coagulopathy development in several studies^25,26^. Notably, the requirement of damage-control surgery was less frequent among patients with immediate CT in this study, whereas frequency of haemostatic surgery was comparable between those with an immediate and non-immediate CT.

Subgroup analyses suggested that older patients and patients with severe injury and decreased consciousness would be ideal candidates for immediate CT. Considering that life-threatening injuries can be misdiagnosed in patients with older age due to distracting pain or disturbed consciousness because of a lack of typical physical signs, whole-body CT can effectively detect injuries in this population^27,28^. Therefore, the favourable effects of immediate whole-body CT would be manifested in these patients. Furthermore, only patients intubated during the initial resuscitation phase benefited from the immediate CT. Obtaining CT images is not a treatment, and therefore the therapeutic effects after immediate CT would be evident only among patients with a considerably severe injury who need any intervention^28^. Intubation at the initial resuscitation phase might be a surrogate marker for injury severity and may be useful to select candidates for immediate CT.

While haemodynamic instability has been suggested as a contraindication for CT, patients with a SBP of less than 90 mmHg on arrival had survival benefits from immediate CT in this study. Although data regarding haemodynamic status before, during, and after CT scanning are unavailable, simultaneous resuscitation in the CT room could have prevented improper management of hypotensive trauma patients^24,29^. Moreover, the association between immediate CT and lower in-hospital mortality was observed regardless of the frequency of immediate CT at each institution. Considering that fewer than 15 hospitals had an all-in-one resuscitation room in Japan^8^, the results in this study suggest that obtaining CT images with a door-to-CT time of 10 min or less would be achieved effectively and safely even in regular trauma centres.

It should also be emphasized that this study does not recommend immediate CT without appropriate resuscitation, including airway management, transfusion, and decompression of tension pneumothorax/cardiac tamponade. It should be emphasized that limited resources of staff and equipment for immediate CT with simultaneous trauma resuscitation would introduce unfavourable outcomes. Moreover, this study only included patients who underwent whole-body CT, and more than a few patients can be urgently transferred to the operating room for haemostatic surgery without detailed CT examination; however, several patients may still need to be evaluated by whole-body CT to identify a potentially life-threatening injury that would not be found on physical examination, X-rays, and ultrasound^30^. Therefore, shortening the door-to-CT time should be considered for severely injured patients with an ISS less than 15 unless emergency surgery is indicated without CT.

The results in this study must be interpreted within the context of the study design. While the results suggest that immediate CT would be effectively achieved even in regular trauma centres without an all-in-one resuscitation room, differences in the availability of resources for trauma resuscitation could affect the results. In addition, the data from the JTDB do not record the indication for the immediate CT (door-to-CT time of 10 min or less). Therefore, these results could have been different if the decision to conduct immediate CT depended on unrecorded strong prognostic factors. Another limitation is that the details of the resuscitative procedure and haemodynamic status before, during, and after CT were not available. Although the initial assessment and resuscitation would follow a protocol similar to ATLS, except for the timing of a whole-body CT, the safety of an immediate CT could not be validated based on objective data. Moreover, only trauma patients with ISS greater than 15 who underwent whole-body CT within 1 h after hospital arrival were investigated, as it is considered that the beneficial effects would influence this population the most. These results cannot be generalized to patients with non-severe injury, needing only selected-region CT, or with no need for CT because of obvious indication for emergency surgery.

Immediate CT was associated with decreased in-hospital mortality and better neurological status at hospital discharge among severely injured trauma patients who underwent head-to-pelvis whole-body CT within 1 h after arrival in hospital. This association was observed regardless of haemodynamic instability on arrival and the frequency of the immediate CT at an institution.

## Supplementary Material

zrac133_Supplementary_Data

## Data Availability

The data of this study are available from the JAST and the Japanese Association for Acute Medicine (JAAM); however, restrictions apply to the data availability, which were used under license for this study and so are not publicly available. Data are available from the authors upon reasonable request with permission from the JAST and the JAAM.
