# Supplementary material for: Immediate CT after hospital arrival and decreased in-hospital mortality in severely injured trauma patients
Source: BJS Open. 2023 Jan 21;7(1):zrac133. doi: 10.1093/bjsopen/zrac133 (PMC9866241; doi:10.1093/bjsopen/zrac133)
Supplement: zrac133_Supplementary_Data [file zrac133_supplementary_data.zip › Supplementary material.docx]

**Immediate Computed Tomography Following Hospital Arrival and Decreased In-hospital Mortality in Severely Injured Trauma Patients**

Ryo Yamamoto, MD, PhD^1^, Masaru Suzuki, MD, PhD^2^, Tomohiro Funabiki, MD, PhD^3^, Junichi Sasaki, MD, PhD^4^

^1^Department of Emergency and Critical Care Medicine, Keio University School of Medicine, Tokyo, Japan, ryo.yamamoto@gmail.com

^2^Department of Emergency Medicine, Tokyo Dental College, Ichikawa General Hospital, Chiba, Japan, suzuki.a2@keio.jp

^3^Department of Emergency Medicine, Fujita Health University Hospital, Aichi, Japan, funabiki@jf7.so-net.ne.jp

^4^Department of Emergency and Critical Care Medicine, Keio University School of Medicine, Tokyo, Japan, sasakij@1989.jukuin.keio.ac.jp

**Ryo Yamamoto, MD, PhD**

**Trauma Service/Department of Emergency and Critical Care Medicine**

**Keio University School of Medicine**

**35 Shinanomachi, Shinjuku, Tokyo 160-8582, Japan**

**Phone: +81-3-3225-1323**

**Fax: +81-3-3353-2232**

**Email:** [**ryo.yamamoto@gmail.com**](mailto:ryo.yamamoto@gmail.com)

**ORCID ID: 0000-0001-8772-9600**

**Supplementary Materials - Index**

| **Supplementary Appendixes** |  |
| --- | --- |
| Appendix S1. Data Source and Data Definitions | *pag. 2* |
| **Supplementary Figures and Tables** |  |
| Figure S1. Box plot of propensity scores before and after weighting | *pag. 3* |
| Table S1. Variance ratio of continuous variables after IPW | *pag. 4* |
| Table S2. In-hospital mortality in sensitivity analyses | *pag. 5* |

**Supplementary Appendixes**

**Appendix S1. Data Source and Data Definitions**

**Data Source:** Japan Trauma Data Bank (JTDB)

JTDB is a nationwide trauma registry established in 2003. The JTDB has been maintained by the Japanese Association for the Surgery of Trauma (JAST) and the Japanese Association for Acute Medicine, representing >250 participating hospitals. There are approximately 300 high-level tertiary care centers in Japan and most participate in the JTDB. Data cleaning is conducted by JAST and JAAM. Data is not linked to any other database.

JTDB is available from the JAST and the JAAM; however, restrictions apply to the data availability, which were used under license for this study and so are not publicly available.

Further details of JTDB are described in the following:

- Japan Trauma Data Bank Annual Report 2021 (2020.1-2020.12) https://www.jtcr-jatec.org/traumabank/dataroom/data/JTDB2021e.pdf
- Yokota J. [Japan Trauma Data Bank (JTDB) managed by Japan Trauma Care and Research (JTCR)]. Nihon Rinsho. 2016 Feb;74(2):329-36. Japanese. PMID: 26915261.
- Hondo K, Shiraishi A, Fujie S, Saitoh D, Otomo Y. In-hospital trauma mortality has decreased in Japan possibly due to trauma education. J Am Coll Surg. 2013 Nov;217(5):850-7.e1.

**Data Definitions (translated to English from Japanese)**

**Inclusion criteria**

≥18 years [Age ≥ 18]

AND

underwent whole-body CT scan [CT scan = “2: whole-body”]

AND

directly transported from the scene [route of transport = “1: from scene”]

**Exclusion criteria**

injury severity score (ISS) <15 [ISS (AIS 2008) < 15]

OR

underwent CT later than one hour after hospital arrival [time from arrival date/time to CT date/time > 1h]

**Supplementary Figures and Tables**

**Fig. S1 Box plot of propensity scores before and after weighting**

**
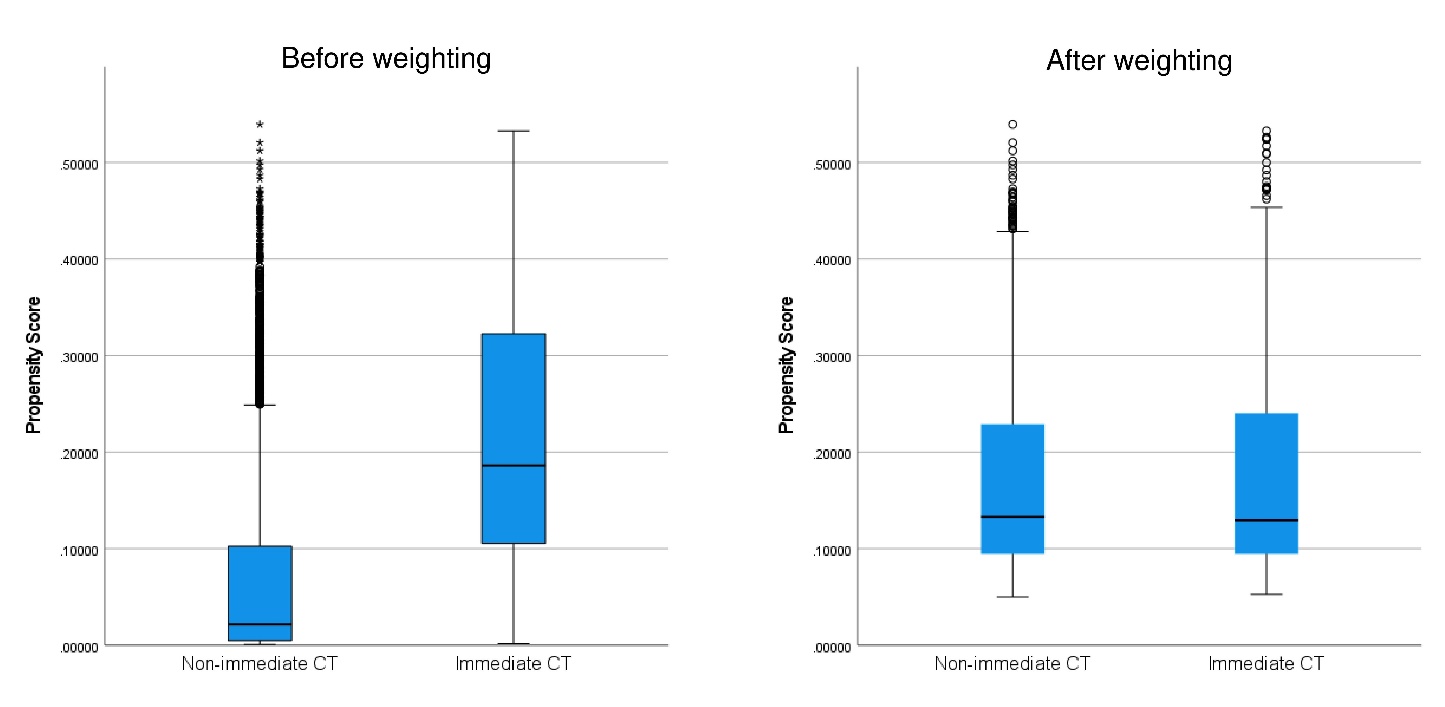
**

| **Table S1. Variance ratio of continuous variables after IPW** | | | | | | | |
| --- | --- | --- | --- | --- | --- | --- | --- |
|  |  | **Immediate CT** | | **Non-immediate CT** | | **Standardized Difference** | **Variance Ratio** |
| **Age, years, median (IQR)** | | **65** | **(50-77)** | **66** | **(47-77)** | **0.001** | **1.036** |
| **Comorbidity, Charlson index, median (IQR)** | | **0** | **(0-0)** | **0** | **(0-1)** | **0.085** | **1.574** |
| **Vital signs on hospital arrival, median (IQR)** | |  |  |  |  |  |  |
|  | **GCS** | **14** | **(10-15)** | **14** | **(8-15)** | **0.069** | **1.281** |
|  | **SBP, mmHg** | **123** | **(100-152)** | **131** | **(105-157)** | **0.090** | **1.270** |
|  | **HR, /min** | **82** | **(68-99)** | **82** | **(69-99)** | **0.041** | **1.283** |
|  | **RR, /min** | **20** | **(18-24)** | **20** | **(17-25)** | **0.017** | **1.460** |
|  | **BT, ℃** | **36.3** | **(35.8-36.8)** | **36.3** | **(35.8-36.7)** | **0.014** | **1.033** |
| **AIS, median (IQR)** | |  |  |  |  |  |  |
|  | **Head** | **2** | **(1-4)** | **2** | **(0-4)** | **0.000** | **1.104** |
|  | **Face** | **0** | **(0-1)** | **0** | **(0-0)** | **0.068** | **0.985** |
|  | **Neck** | **0** | **(0-0)** | **0** | **(0-0)** | **0.031** | **0.917** |
|  | **Chest** | **2** | **(0-3)** | **1** | **(0-3)** | **0.012** | **1.103** |
|  | **Abdomen/pelvis** | **0** | **(0-0)** | **0** | **(0-0)** | **0.000** | **1.294** |
| **RTS** |  | **7.55** | **(6.17-7.84)** | **7.84** | **(5.97-7.84)** | **0.006** | **1.311** |
| **IPW = inverse probability weighting, CT = computed tomography, IQR = interquartile range, GCS = Glasgow Coma Scale, SBP = systolic blood pressure, HR = heart rate, RR = respiratory rate, BT = body temperature, AIS = abbreviated injury scale, and RTS = revised trauma score.** | | | | | | | |
|  |  |  |  |  |  |  |  |
|  |  |  |  |  |  |  |  |
|  |  |  |  |  |  |  |  |

| **Table S2. In-hospital mortality in sensitivity analyses** | | | | |
| --- | --- | --- | --- | --- |
|  | **Immediate CT** | **Non-immediate CT** | **OR** | **95% CI** |
| **In-hospital mortality, *% (95% CI)*** |  |  |  |  |
| **Multivariate logistic regression*** |  |  | **0.67** | **0.46–0.97** |
| **Generalized estimating equations** |  |  | **0.69** | **0.49–0.99** |
| **IPW with restriction**** | **13.7 (12.4–15)** | **16.5 (15.2–17.9)** | **0.81** | **0.69–0.93** |
| **CT= computed tomography, OR = odds ratio, CI = confidence interval, and IPW = inverse probability weighting. *Final model included immediate CT, sex, mechanism of injury, Charlson comorbidity index, respiratory rate, Abbreviated Injury Scale in the head and chest, prehospital oxygen and fluid administration, presence of physician at prehospital, and the frequency of immediate CT. **Patients with propensity score >0.05 and <0.95 were included.** | | | | |
